# Editorial: Hormone resistance in cancer

**DOI:** 10.3389/fendo.2023.1272932

**Published:** 2023-08-24

**Authors:** John T. Phoenix, Audris Budreika, Raymond J. Kostlan, Justin H. Hwang, Sean W. Fanning, Steven Kregel

**Affiliations:** ^1^ Department of Cancer Biology, Stritch School of Medicine, Loyola University Chicago, Maywood, IL, United States; ^2^ Integrated Program in Biomedical Science, Biochemistry, Molecular and Cancer Biology, Loyola University Chicago, Maywood, IL, United States; ^3^ Department of Medicine, University of Minnesota, Minneapolis, MN, United States

**Keywords:** neuroendocrine, nuclear hormone receptors, hormone signaling, breast, prostate, lineage plasticity, pituitary

## Introduction

Hormone-dependent cancers are the most common non-cutaneous tumors experienced by all sexes. This year, nearly 700,000 combined new cases of breast, ovarian, endometrial, prostate, and thyroid cancers will be diagnosed in the United States (1). Antagonizing hormone signaling pathways is a widely used treatment strategy (2–6). However, due to acquired mutations of hormone receptors, indirect cofactor-mediated changes in cell behavior, and heterogeneity within tumors, the therapeutic durability of these treatments is often short-lived (7–13). In late-stage disease, a lethal, intractable small-blue cell tumor or neuroendocrine-like phenotype may emerge displaying genetic, epigenetic, and hormonal properties promoting cellular plasticity (11, 12, Imamura et al., 14). Specific neuroendocrine tumor features are poorly defined and vary across tissue origin (14, 15). Therefore, understanding these mechanisms of treatment resistance and finding commonalities among neuroendocrine subsets of cancer is vital to uncover new targeted therapies beyond hormone therapy that halt disease progression.

## Hormones and cancers

### Hormone receptor signaling

Hormones often serve as activating ligands for their respective nuclear hormone receptor (NHR) proteins: Estrogen Receptor (ER) in breast, ovarian, and endometrial tissue, and Androgen Receptor (AR) in prostate tissue (16, 17). Thyroid carcinomas are often fueled by Thyroid–Stimulating Hormone (TSH) produced in the pituitary gland, responsible for the endocrine secretion of many hormones (Mousa et al.). NHRs act as potent activators of oncogenes in transformed cells (4, 17). Ultimately, maintenance of hormone signaling pathways despite attempted blockades is a mechanism of disease progression (18–20).

### Hormone therapy in cancer

Targeting hormone signaling pathways are a clinical mainstay for treating hormone–dependent cancers. Exogenous L–Thyroxine (T4) is a common treatment to promote a negative feedback loop of TSH signals in papillary and follicular thyroid cancers (Mousa et al.). Similarly, gonadotropin releasing hormone (GnRH) antagonists, or agonists through feedback, castrate prostate cancer patients by halting testicular production of androgens (21). Androgen and estrogen biosynthesis inhibitors, abiraterone acetate and aromatase inhibitors, respectively, inhibit enzymes that synthesize hormones in patients (22, 23). Second–generation AR antagonists like enzalutamide and darolutamide impede AR interaction with testosterone (24). ER–targeted therapies include the Selective Estrogen Receptor Modulators/Degraders (SERM/Ds). SERMs tamoxifen and raloxifene bind to the ER ligand binding domain and obstruct ER signaling breast tissue (25, 26). Selective Estrogen Receptor Degraders (SERDs) bind and target ER for proteasomal degradation (27, 28).

### Hormone therapy resistance

Unfortunately, the response to hormone therapy is temporary. As discussed in Mousa et al., L–Thyroxine may in fact stimulate tumor cell proliferation in patients with therapy–resistant thyroid cancer. Breast and prostate tumors often exhibit NHR mutations that render most treatments ineffective, including activating mutations where enzalutamide and tamoxifen can serve as functional NHR ligands. (7, 8, 20, 29–33). Cancer cells employ growth pathways aside from NHR signaling and utilize alternative cofactors and coregulatory molecules promoting disease progression (2, 9, 10, 34–38). Since NHR family proteins are structurally similar, other NHRs such as Glucocorticoid Receptor (GR) can compensate for loss of AR/ER activity (39, 40). These resistance mechanisms allow for initially hormone–driven tumors to become metastatic, hormone–indifferent disease (18). Imamura et al. found these cancers often display a high degree of lineage–plasticity and sometimes complete loss of NHR–dependence in Neuroendocrine Prostate Cancer (NEPC) and Triple–Negative Breast Cancer (TNBC) (41).

## Neuroendocrine differentiation and hormone independence

### Neuroendocrine tumor characteristics

Since very few neuroendocrine molecular markers exist across tumor types, specifically defining these populations and targeting these poorly differentiated, aggressive cancer cells remains elusive. Neuroendocrine tumors typically display markers of neuronal differentiation and can originate in various anatomical locations. Nevertheless, they exhibit histological and clinical resemblances (42). Neuroendocrine subsets seen in late–stage cancers are poorly differentiated, with large nuclear–to–cytoplasmic ratios, that aberrantly activate stem cell gene pathways (34, 43, 44).

**Figure 1:**
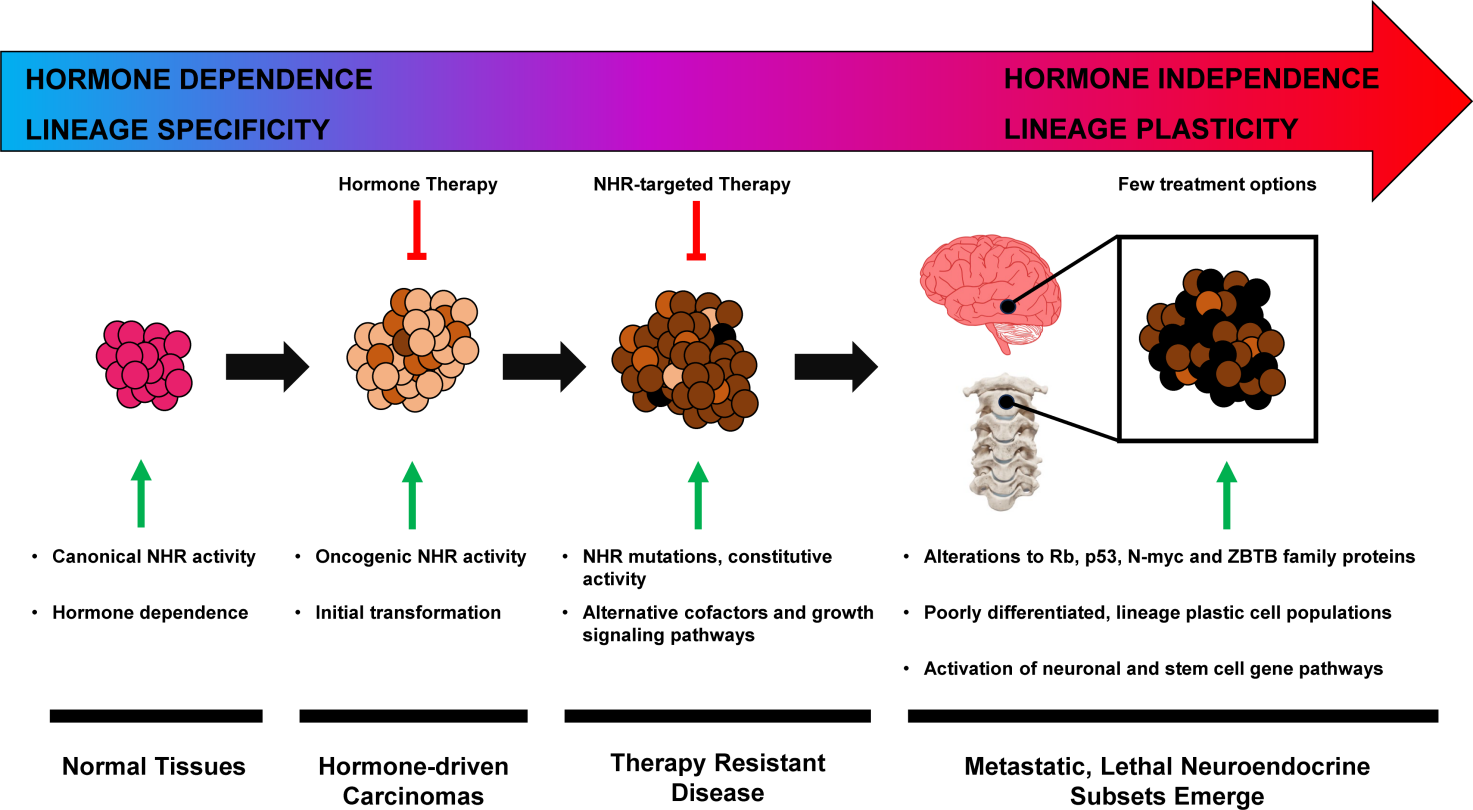
Hormone–driven cancer progression to neuroendocrine phenotype.

Some genetic similarities are shared across various small–cell neuroendocrine (SCN) diseases such as small–cell lung cancer (SCLC) and NEPC, regardless of their tissue of origin (44). These can include *RB1* deletion, *TP53* mutation, and *N–MYC* overexpression (42, 44–47). In hormone dependent tumors, *de novo* incidence of SCN/NEPC is rare, and most cases result from therapeutic pressure (48, 49). Further complicating the topic is that neuroendocrine neoplasms occur in many sites: the central nervous system, respiratory tract, gastrointestinal tract, thyroid, breast, and urogenital system, and yet share similar pathologic features (50). Although progress has been made in the management of these lineage plastic neuroendocrine cancers, such as Delta–like–ligand–3 (DLL3) targeted molecules (51, 52), more research is needed to further characterize the exact molecular mechanisms of progression to an SCN phenotype (**Figure 1**).

### Emerging factors in SCN tumors

A recently developed SCN phenotype grading system was used by Bae et al. to demonstrate oncogenic function of the transcription factor ZBTB7A in NEPC (42). ZBTB (Zinc finger and broad–complex, tramtrack and bric–a–brac domain containing) protein family members are multifunctional transcription factors that play significant roles cell proliferation, differentiation, and development. (53, 54). Elevated expression of ZBTB7A has been linked to tumor formation and metastasis in different cancer types, including breast, prostate, lung, ovarian, and colon cancer (55, 56). Interestingly, pituitary neuroendocrine tumors (PitNETs) are characterized by overexpression of prolactin, Studies have demonstrated that ZBTB20 plays a role in controlling prolactin expression in the pituitary gland and contributes to the development of hepatocellular carcinoma (57–59). Lin et al. describe a patient in whom a PitNET had developed resistance to standard of care therapy was treated with hydroxychloroquine and experienced a significant reduction in prolactin secretion. Given the established crosstalk between prolactin and estrogen receptor signaling in breast cancer, ZBTB family transcription factors could play key roles in the emergence and therapy resistance neuroendocrine cancers (37, Bae et al., 60). These factors may drive lineage–plasticity across tumor types and the loss of NHR–dependence that characterizes SNC/NEPC.

## Summary

Hormone driven cancers are common malignancies that sometimes differentiate into lethal, aggressive neuroendocrine subtypes. In breast and prostate cancer, neuroendocrine differentiation often emerges after the failure of hormone targeted therapies. Currently, a limited number of dependable neuroendocrine molecular markers exist across tissues. Further research is required to discover genetic similarities that can pave the way for effective targeted therapies capable of eradicating multiple subtypes of neuroendocrine and lineage plastic tumors.

## Author contributions

JP: Conceptualization, Writing – original draft, Writing – review & editing. AB: Conceptualization, Writing – original draft, Writing – review & editing. RK: Conceptualization, Writing – original draft, Writing – review & editing. JH: Conceptualization, Writing – original draft, Writing – review & editing. SF: Conceptualization, Writing – original draft, Writing – review & editing. SK: Conceptualization, Project administration, Supervision, Validation, Writing – original draft, Writing – review & editing.
